# HPLC Analysis of Phenolic Compounds and Flavonoids with Overlapping Peaks

**DOI:** 10.17113/ftb.58.01.20.6395

**Published:** 2020-03

**Authors:** Luke Mizzi, Christina Chatzitzika, Ruben Gatt, Vasilis Valdramidis

**Affiliations:** 1 Department of Food Studies and Environmental Health, Faculty of Health Sciences, University of Malta, Triq il-Qroqq, Msida MSD 2080, Malta; 2 Metamaterials Unit, Faculty of Science, University of Malta, Triq il-Qroqq, Msida MSD 2080, Malta

**Keywords:** HPLC analysis, UV-Vis absorbance, quantification of phenolic compounds and flavonoids, overlapping peaks

## Abstract

The identification and quantification of phenolic compounds and flavonoids in various natural food products is typically conducted using HPLC analysis. Their analysis is particularly complex since most natural food products contain a large number of different phenolic compounds, many of which have similar chemical characteristics such as polarity, which makes complete separation of all eluents extremely difficult. In this work we present and validate a method for the quantitative determination of the concentration of two compounds with similar retention times, *i.e.* they show overlapping peaks in a mixed solution. Two pairs of phenolic compounds were investigated: caffeic and vanillic acids and ferulic and *p*-coumaric acids. This technique takes advantage of the different absorbances of the two phenolic compounds in the eluent at various wavelengths and can be used for the quantitative determination of the concentration of these compounds even if they are not separated in the HPLC column. The presented method could be used to interpret the results of HPLC analysis of food products which possess a vast spectrum of phenolic compounds and flavonoids.

## INTRODUCTION

Phenolic compounds and flavonoids are a class of natural products commonly found in food products (*1*-*4*) including herbs (*5*), wine (*6*-*8*), beer (*9*), olive oil (*10*-*13*), fruits (*14*, *15*) and honey (*16*-*20*). Despite being present in relatively small concentrations, these compounds are known to impart beneficial properties to these food products such as antimicrobial, food preservation and antioxidant properties (*8*, *17*, *19*, *21*-*27*). The amount and type of these compounds depends primarily on the product type and location, and in the case of honey, floral sources, so they can also sometimes serve as chemical fingerprints to trace the geographic and botanical origins of the food products.

The identification and quantification of phenolic compounds and flavonoids in food products is typically conducted using HPLC analysis with a UV-Vis diode array detector (DAD) (*7*, *25*, *28*-*37*). The regular *modus operandi* involves the isolation and extraction of phenolic compounds from the food product, followed by an HPLC run using a gradient mobile phase consisting of two or more reagents, which are typically a polar organic solvent such as methanol or acetonitrile and a weak acid such as phosphoric or acetic acid (*30*, *31*, *38*). The analytes are then identified and quantified by comparison against standard solutions. While this method is perfectly valid and accurate for certain food products, it may however prove to be insufficient for the analysis of products such as olive oil, wine and honey, which contain a considerably large assortment of natural products, most of which are chemically related and have similar polarity. This can make separation of peaks problematic, resulting in some cases in amalgamated peaks, which makes it difficult to determine the exact concentration of certain compounds, or indeed, in some situations, even to simply ascertain their presence in food products, particularly if most of the peaks in the spectrum are unidentified. In such scenarios, it is extremely unlikely that an analysis based solely on a single HPLC spectrum is sufficient to obtain a completely accurate and reliable characterization and quantification of these compounds.

In view of this, the objective of this work is to propose a method that can be used to identify and quantify with a high degree of certainty fifteen phenolic compounds commonly found in a variety of natural food products ranging from honey and olive oil to fruit juices. The specific aim is the determination of the concentration of phenolic compounds that have overlapping peaks by taking advantage of their diverse absorbances at different wavelengths. Accurate determination of the individual concentrations of phenolic compounds having peaks with identical retention times in a mixture is the ultimate objective.

## MATERIALS AND METHODS

### Preparation of standards and phenolic mixtures

Standard solutions were prepared for the fifteen investigated phenolic compounds and flavonoids, namely: kaempferol, luteolin, phenylacetic acid (all Alfa Aesar, Ward Hill, MA, USA), apigenin, chrysin, quercetin, *p*-coumaric acid, naringenin (all Sigma-Aldrich, Merck, Darmstadt, Germany), ferulic, syringic, vanillic, caffeic, ellagic, gallic and benzoic acids (Acros Organics, Geel, Belgium). The solutions were prepared by dissolving the standards in HPLC grade methanol (ultragradient grade; Carlo Erba, Milan, Italy) to produce stock solutions of 100 mg/L, which were then used to prepare 50, 40, 30, 20 and 10 mg/L solutions for the standard plots. In addition, a mixture containing 30 mg/L of each phenolic compound in methanol was also prepared. Two mixtures of *p*-coumaric and ferulic acid were prepared, one with equal concentrations of 50 mg/L each and the other with 30 mg/L *p*-coumaric and 70 mg/L ferulic acid. Another similar set of mixtures was also prepared using vanillic and caffeic acids. We measured the absorbance of the samples of 100 mg/L of each phenolic compound and flavonoid with a UV-Vis spectrophotometer (UV-2600; Shimadzu, Tokyo, Japan) at wavelengths between 180 and 480 nm to find the optimum wavelength for the HPLC-DAD measurements.

### HPLC analysis

The HPLC analysis of the phenolic compounds and flavonoids was conducted using a Waters 2695 Alliance HPLC system (Waters Inc., Milford, CT, USA), equipped with a UV-Vis DAD. The separation was conducted using a Waters Sunfire^TM^ C18 reverse-phase chromatography column, 250 mm length, 4.6 mm width, and particle size 5 μm. The phenolic standard solutions and mixtures were injected into the system using an autoinjector. Different isocratic and gradient mobile phases were tested at different flow rates and column temperatures in order to find a suitable separation method for the standards.

The gradient method that was eventually chosen following a series of preliminary studies uses a mixture of acetonitrile (mobile phase A, HPLC grade ≥99.9%; Honeywell Seelze, Germany) and phosphoric acid (mobile phase B), which was prepared by dropwise addition of 85% orthophosphoric acid (Sigma-Aldrich, Merck, Darmstadt, Germany) to HPLC grade water (Carlo Erba) until pH=2 was reached. The total runtime of the method was 60 min and the concentration gradient was varied as follows: a) initially 5% A and 95% B, b) 15 min 35% A and 65% B, c) 20 min 35% A and 65% B, d) 30 min 40% A and 60% B, e) 35 min 40% A and 60% B, f) 40 min 50% A and 50% B, g) 52 min 70% A and 30% B and h) 60 min 5% A and 95% B. A constant flow rate of 0.5 mL/min and a temperature of 5 °C were used. Following the analysis of the UV-Vis spectra of the individual phenolic standards, three wavelengths (210, 280 and 360 nm) were chosen for analysis in this investigation using the HPLC-DAD.

## RESULTS AND DISCUSSION

**Fig. 1:**
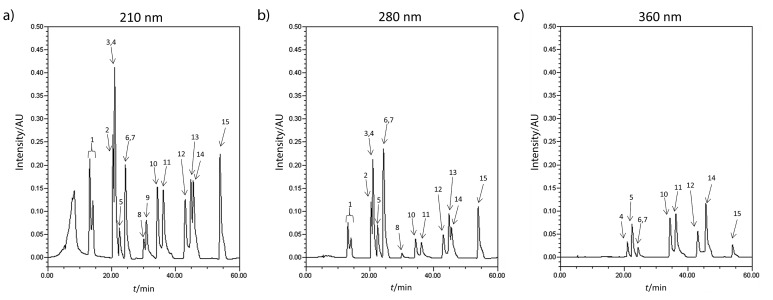
Chromatograms showing the peaks obtained for the mixture containing all 15 phenolic compounds (*γ*=30 mg/L) at: a) *λ*=210 nm, b) *λ*=280 nm and *λ*=360 nm. The peaks represent the following phenolics: 1=gallic acid, 2=syringic acid, 3=caffeic acid, 4=vanillic acid, 5=ellagic acid, 6=*p*-coumaric acid, 7=ferulic acid, 8=benzoic acid, 9=phenylacetic acid, 10=luteolin, 11=quercetin, 12=apigenin, 13=naringenin, 14=kaempferol and 15=chrysin. Note that not all phenolic compounds show peaks at all of the three wavelengths tested and that caffeic and vanillic acids (3 and 4) and *p*-coumaric and ferulic acids (6 and 7) show only one joined peak each at retention time *t*_R_=21.0 and 24.5 min respectively

Fig. 1 shows the chromatograms of the solution containing all 15 phenolic compounds obtained at wavelengths of 210, 280 and 360 nm. Table 1 and Table 2 show the retention times and calibration constants based on the area and height of peaks for each phenolic compound for every wavelength, respectively.

**Table 1:** Retention times, *t*_R_, absorbance constants, *k* and *b*, and coefficient of determination, R^2^, at *λ*=210, 280 and 360 nm based on the area under the peak obtained through numerical integration

Phenolic compound	*t*_R_/min	*k*_210 nm_/(L/mg)	*b*_210 nm_	R^2^_210 nm_	*k*_280 nm_/(L/mg)	*b*_280 nm_	R^2^_280 nm_	*k*_360 nm_/(L/mg)	*b*_360 nm_	R^2^_360 nm_
Luteolin	34.6	362968	-520799	0.9957	97486	-242167	0.9953	194425	-184215	0.9957
Gallic acid	13.2	459617	-770358	0.9971	166881	-254698	0.9968	N/A	N/A	N/A
Benzoic acid	30.3	86368	20923	0.9996	20040	6292	0.9995	N/A	N/A	N/A
Ferulic acid	24.4	130912	11975	0.9997	119049	-27757	0.9991	22754	3712.8	0.9991
Caffeic acid	21.0	149934	509654	0.9992	127745	138030	0.9990	31426	44101	0.9996
Chrysin	54.1	458102	-336458	0.9924	225016	-136314	0.9928	54173	-37189	0.9922
*p*-Coumaric acid	24.2	139489	-28871	0.9931	197775	-148970	0.9945	2767	4959	0.9933
Vanillic acid	20.9	222003	189562	0.9959	63869	-25999	0.9907	N/A	N/A	N/A
Ellagic acid	22.4	78174	-125923	0.9983	87867	-83342	0.9980	87512	-79105	0.9982
Apigenin	43.0	246136	748603	0.9985	101424	325117	0.9985	111217	377999	0.9987
Kaempferol	45.6	221097	-287675	0.9953	75058	-93253	0.9954	196601	-225360	0.9955
Syringic acid	20.3	326318	-797339	0.9965	156888	-626034	0.9974	N/A	N/A	N/A
Naringenin	44.7	139804	49990	0.9930	5307.3	4973.9	0.9948	N/A	N/A	N/A
Quercetin	36.1	266832	-481865	0.9491	163593	-382709	0.9721	54267	-44453	0.9576
Phenylacetic acid	31.0	131438	-259598	0.9933	N/A	N/A	N/A	N/A	N/A	N/A

N/A=not available

**Table 2:** Retention times, *t*_R_, absorbance constants *k* and *b*, and coefficient of determination, R*^2^*, at *λ*=210, 280 and 360 nm based on peak height

Phenolic compound	*t*_R_/min	*k*_210 nm_ /(L/mg)	*b*_210 nm_	R^2^_210 nm_	*k*_280 nm_ /(L/mg)	*b*_280 nm_	R^2^_280 nm_	*k*_360 nm_/(L/mg)	*b*_360 nm_	R^2^_360 nm_
Luteolin	34.6	9753.4	-37657	0.9736	2556.5	-10098	0.9740	5292.9	-20850	0.9735
Gallic acid	13.2	8971.7	-18458	0.9965	3262.1	-6646.3	0.9964	N/A	N/A	N/A
Benzoic acid	30.3	2350.9	536.5	0.9997	546.47	41	0.9998	N/A	N/A	N/A
Ferulic acid	24.4	8712.3	1990.7	0.9976	8025.4	-2626.4	0.9977	1532.11	63.344	0.9976
Caffeic acid	21.0	11765	14880	0.9860	10504.8	9830.6	0.9911	2569.53	2860.9	0.9755
Chrysin	54.1	11362	-12923	0.9994	5615.7	-6468	0.9995	1348	1518	0.9995
*p*-Coumaric acid	24.2	9232.5	-2771.8	0.9723	13161	-11585	0.9994	176.212	366.92	0.9992
Vanillic acid	20.9	17468	3311.6	0.9933	5035.7	-2175.2	0.9933	N/A	N/A	N/A
Ellagic acid	22.4	1944.6	3959.5	0.9920	2164	4922.6	0.9919	1944.6	3959.5	0.9920
Apigenin	43.0	4421.7	17239	0.9973	1174.2	6967.6	0.9973	1965.2	7791.1	0.9973
Kaempferol	45.6	4912.6	-6632.2	0.9954	1669	-2265.3	0.9953	4383.3	-5923.6	0.9953
Syringic acid	20.3	11132	9202.5	0.9900	5124.1	6733.2	0.9906	N/A	N/A	N/A
Naringenin	44.7	3470.5	-6885.7	0.9963	127.62	-142.6	0.9969	N/A	N/A	N/A
Quercetin	36.1	4983.7	-2190	0.9475	3075	854.5	0.9699	1065.7	117.9	0.9705
Phenylacetic acid	31.0	2980.3	4611.5	0.9979	N/A	N/A	N/A	N/A	N/A	N/A

N/A=not available

As one can observe from the chromatograms in Fig. 1, the gradient method used here separates most phenolic compounds reasonably well with most of them showing distinct and sharp individual peaks. Moreover, while all phenolic compounds show peaks at 210 and 280 nm (except for phenylacetic acid at 280 nm), luteolin, ferulic acid, caffeic acid, *p*-coumaric acid, ellagic acid, apigenin, kaempferol and quercetin also show peaks at 360 nm. These results are in accordance with those obtained from the initial tests conducted using a UV-Vis spectrophotometer to determine the choice of wavelengths.

However, as one may observe in Fig. 1, there are also two pairs of phenolic compounds which have identical retention times and, hence overlapping peaks: vanillic and caffeic acids at 21.0 min and ferulic and *p*-coumaric acids at 24.5 min. Yet this drawback does not necessarily mean that it is impossible to determine the individual concentrations of these phenolic compounds. As evident from the values in Table 1 and Table 2, each phenolic compound has a different absorption profile. It is possible to take advantage of this property to determine the concentration of each phenolic compound in the mixture by using the standardization constants of the individual phenolic compounds and the total absorbance of the phenolic mixture at different wavelengths.

The method proposed here operates under the assumption that the total area of the peak at a given wavelength is equal the sum of the individual areas of the phenolic compounds, making up the peak, Ph_i_ and Ph_j_, at the same wavelength, *λ*_i_:


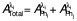


This relationship is valid for all wavelengths and thus Eq. 1 can be used to generate the following simultaneous equations for the peaks obtained at two different wavelengths:
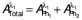
and


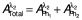


These equations can be expanded to incorporate the terms defining the concentrations (gamma Ph1) of the phenolic compounds and the standardization gradient and y-intercept constants, which are related to the area, through the following equation:
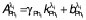
thus:
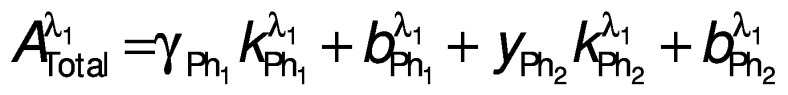
and


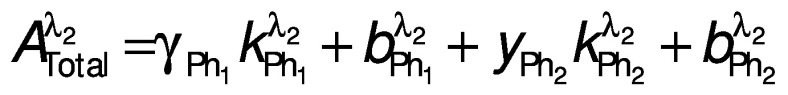


As one may observe from Eqs. 5 and 6, the terms and are common for both equations and thus, since all the other terms are known, one may obtain the values for these concentrations by solving the two simultaneous equations. The final values for and may be expressed as follows:
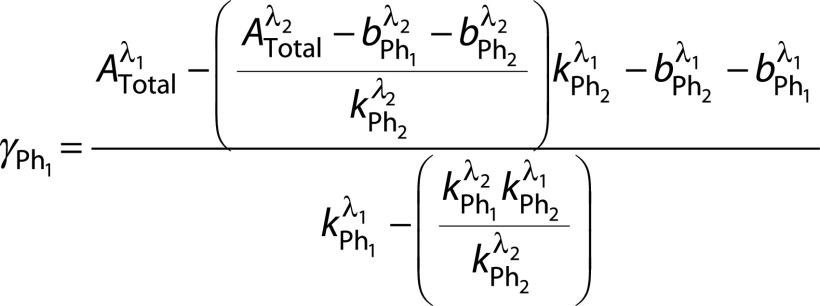
and


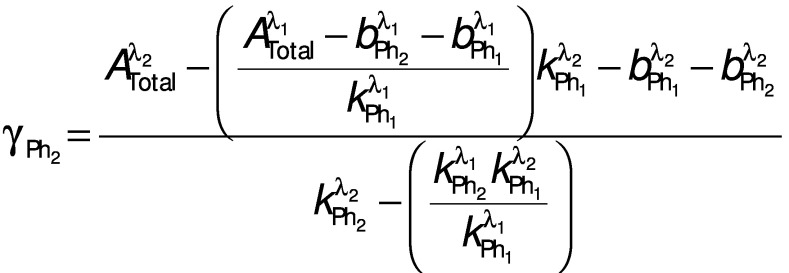


These equations may be used to calculate the concentrations of *p*-coumaric and ferulic acids since these two phenolics have very similar retention times and absorb to different extents at all of the three wavelengths used here. In the case of vanillic and caffeic acids, the problem is simpler since while the latter absorbs at all three wavelengths, the former absorbs only at *λ*=210 and 280 nm. Therefore, Eqs. 7 and 8 may be simplified as follows to calculate the concentrations of these phenolics when considering a wavelength of 210 or 280 nm in conjunction with the 360 nm wavelength, since in the latter case A lambda 1 total is equal to A lambda 2 Ph_1_:
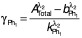
and
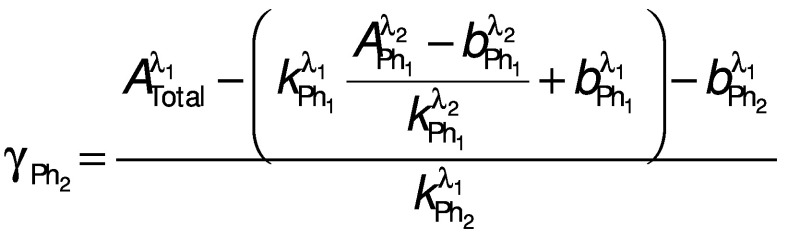
where Ph_1_ is the phenolic compound that absorbs at both evaluated wavelengths, in this case caffeic acid, Ph_2_ is the other, *i.e.* vanillic acid, *λ*_1_ is the wavelength at which both phenolics absorb, in this case 210 or 280 nm, and *λ*_2_ is the wavelength at which only one phenolic compound absorbs, in this case 360 nm.

**Fig. 2:**
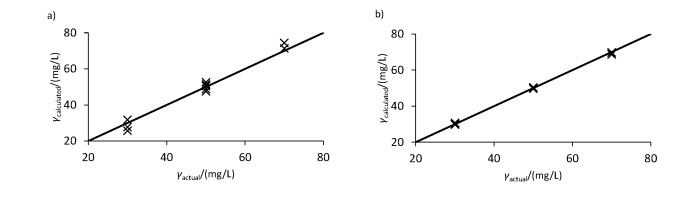
Comparison of the actual and calculated concentrations using Eqs. 7-10 shown in Table 3 based on the peak area for the mixtures of: a) caffeic and vanillic acids (mixtures 1 and 2) and b) ferulic and *p*-coumaric acids (mixtures 3 and 4). The straight black line indicates the point at which the calculated and actual concentrations are equal

In order to validate the effectiveness of this method, Eqs. 7-10 were applied to determine the concentrations of two mixtures of vanillic and caffeic acids (mixtures 1 and 2) and ferulic and *p*-coumaric acid (mixtures 3 and 4) with known concentrations. The concentrations of these mixtures were calculated using the peak areas from three data sets of wavelengths: 210-280, 210-360 and 280-360 nm and the results are presented in Table 3. A comparison between the real and the calculated concentrations of the mixtures is also shown in Fig. 2.

**Table 3:** Concentrations of the two phenolic compounds with similar retention times in a mixture that were experimentally measured and calculated using the peak area method

Mixture	*λ*_1_/nm	*λ*_2_/nm	*A*_lambda 1 total>_	*A*_lambda 2 total>_	*γ*_actual_/(mg/L)		*γ*_calculated_/(mg/L)	
Caffeic acid	Vanillic acid	Caffeic acid	Vanillic acid
1	210	280	19094066	9844450	50	50	52.48	47.42
	210	360	19094066	1631556	50	50	50.51	48.74
	280	360	9844450	1631556	50	50	50.51	51.35
2	210	280	17541467	11247709	70	30	74.34	25.66
	210	360	17541467	2284079	70	30	71.28	27.73
	280	360	11247709	2284079	70	30	71.28	31.79
					Ferulic acid	*p*-Coumaric acid	Ferulic acid	*p*-Coumaric acid
3	210	280	13511557	15649025	50	50	50.41	49.67
	210	360	13511557	1284938	50	50	50.00	50.06
	280	360	15649025	1284938	50	50	50.02	49.91
4	210	280	13268465	14087077	70	30	68.70	30.77
	210	360	13268465	1678385	70	30	69.76	29.77
	280	360	14087077	1678385	70	30	69.71	30.16

It is evident from the data in Table 3 and the plot in Fig. 2 that the values obtained through the equations are extremely similar to the actual concentrations of the individual phenolic acids making up each of the four mixed solutions. In fact, in the case of the mixtures of *p*-coumaric and ferulic acids, the calculated values were all within ±0.5 mg/L of the actual values, indicating a high degree of accuracy. On the other hand, in the cases of vanillic and caffeic acid mixtures, there are slightly more discrepancies between the points, although overall the average predictions of each combination of wavelengths are still very close to the actual values.

**Fig. 3:**
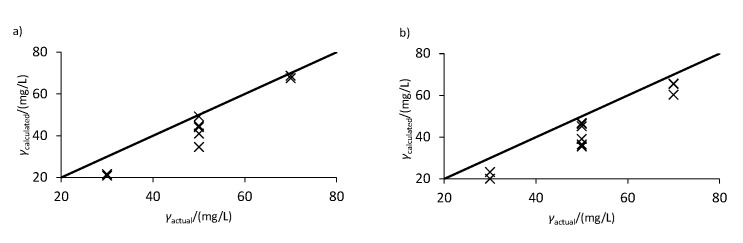
Comparison of the actual and calculated concentrations using Eqs. 7-10 based on the peak height shown in Table 4 for the mixtures of: a) caffeic and vanillic acids (mixtures 1 and 2) and b) ferulic and *p*-coumaric acids (mixtures 3 and 4). The straight black line indicates the point at which the calculated and actual concentrations are equal

These results confirm the validity of Eqs. 7-10 for calculating the concentrations of HPLC analytes with overlapping peaks based on their varying absorbances at different wavelengths. In theory, such a technique should also be applicable to peak height and peak area; however, this is only the case if the phenolic compounds in question possess exactly the same retention times. In the cases presented here the two pairs of phenolic compounds have extremely similar but not exact retention times. This means that while a single large peak is obtained for the mixture, it is wider as well as higher than the individual peaks and thus while the cumulative peak areas of the individual phenolic compounds conform to the assumption presented in Eq. 1, the same cannot be said for the cumulative peak heights:
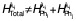
where *H* represents the peak height. In fact, this is evident from the results presented in Table 4 and Fig. 3, where calculations corresponding to Eqs. 7-10 but based on peak height are presented. As one may observe, the calculated values obtained with this method consistently underestimate the phenolic concentration by a large extent, hence confirming the inadmissibility of this method when applied to peak height data.

**Table 4:** Concentrations of two phenolic compounds in a mixture with similar retention times that were experimentally measured and calculated using the peak height (*H*) method

Mixture	*λ*_1_/nm	*λ*_2_/nm	*H*_lambda 1 total>_	*H*_lambda 2 total>_>	*γ*_actual_/(mg/L)		*γ*_calculated_/(mg/L)	
Caffeic acid	Vanillic acid	Caffeic acid	Vanillic acid
1	210	280	1316501	700549	50	50	44.79	44.16
	210	360	1316501	129553	50	50	49.31	41.12
	280	360	700549	129553	50	50	49.31	34.74
2	210	280	1193500	827755	70	30	67.66	21.71
	210	360	1193500	179699	70	30	68.82	20.93
	280	360	827755	179699	70	30	68.82	19.29
					Ferulic acid	*p*-Coumaric acid	Ferulic acid	*p*-Coumaric acid
3	210	280	735240	874422	50	50	36.54	45.24
	210	360	735240	78460	50	50	46.84	35.52
	280	360	874422	78460	50	50	46.42	39.22
4	210	280	740386	776925	70	30	60.40	23.28
	210	360	740386	104352	70	30	65.73	18.25
	280	360	776925	104352	70	30	65.51	20.17

At this point it is important to mention the advantages of using the method described here to analyze UV-Vis HPLC-DAD results. Although typically it is important to ensure that all the analytes separate completely, this is not always so easily achieved, particularly in the case of natural food products such as honey (also evident from previous works (*39*-*41*)), which are known to contain over fifty different types of phenolic compounds and flavonoids. In such cases, finding a gradient method which is capable of achieving complete separation of all constituents is almost impossible, especially since many of these phenolic compounds have extremely similar chemical composition and polarities. By using the method presented here one may possibly circumvent this problem, particularly if like in the case described here, the gradient method is capable of completely separating the majority of phenolic compounds, and therefore there is no need to develop another method solely to separate a couple of peaks. Moreover, the equations described in this methodology can also be used to conduct a qualitative analysis in order to determine if any unknown compounds have overlapping peaks with the target compounds under analysis. If using the equations to calculate the concentrations of two phenolic compounds over multiple pairs of wavelength combinations results in different calculated values, then this is indicative of the presence of possibly a third, unknown eluent contributing to the peak area. On the other hand, if all combinations of wavelengths return the same concentrations, then this confirms that only the two phenolics in question are present at this retention time. Currently, the standard method used to counteract this problem is to either use multiple UV-Vis absorption-based HPLC protocols with different gradient methods and/or mobile phases such as that employed by Gupta *et al*. (*35*), or else to validate the initial HPLC results using additional detectors such as a mass spectrometer (*39*, *42*-*44*). The method proposed in this work eliminates the need of using such techniques as a validation method for a UV-Vis absorption-based HPLC analysis. This would facilitate the analysis of complex solutions since all the results required for this analysis may be obtained from a single HPLC run. However, it should be emphasized that the technique proposed here would replace these techniques for validation and quantification purposes only, and that the use of additional methods such as MS-HPLC is still required for the eventual characterization and identification of any unknown compounds in natural products. Furthermore, this technique could also be potentially employed as a quality control method for the analysis of synthetic products containing phenolic compounds and flavonoids. In such cases where the constituents are already known, a partial HPLC separation coupled with the method applied here could be sufficient to quantify the individual phenolic compound content.

## CONCLUSION

In this work, we presented and validated an HPLC analysis method that can be used to find the concentrations of eluents with similar retention times in a mixture. The analysis was conducted on a mixture of fifteen phenolic compounds, with two pairs of phenolic compounds having peaks with nearly identical retention times, using UV-Vis absorbance measurements from an HPLC-DAD. The results obtained from the equations used to calculate the concentrations based on the peak area standardization constants of the individual phenolic compounds showed excellent agreement with the known concentrations of the mixtures and indicated that this technique could be a viable method to quantitatively analyze the concentrations of such eluents. It is envisaged that this technique could be applied for HPLC analysis of food products such as olive oil, fruit juices and honey, which have a vast spectrum of phenolic compounds and flavonoids with similar chemical characteristics and thus yield complex chromatograms that are extremely difficult to interpret accurately.
